# The effect of brain size evolution on feeding propensity, digestive efficiency, and juvenile growth

**DOI:** 10.1111/evo.12784

**Published:** 2015-10-19

**Authors:** Alexander Kotrschal, Alberto Corral‐Lopez, Sönke Szidat, Niclas Kolm

**Affiliations:** ^1^ Department of Ethology/ZoologyStockholm UniversitySweden; ^2^ Department of Chemistry and BiochemistryUniversity of BernSwitzerland

**Keywords:** Behavior, development, artificial selection, trade‐offs

## Abstract

One key hypothesis in the study of brain size evolution is the expensive tissue hypothesis; the idea that increased investment into the brain should be compensated by decreased investment into other costly organs, for instance the gut. Although the hypothesis is supported by both comparative and experimental evidence, little is known about the potential changes in energetic requirements or digestive traits following such evolutionary shifts in brain and gut size. Organisms may meet the greater metabolic requirements of larger brains despite smaller guts via increased food intake or better digestion. But increased investment in the brain may also hamper somatic growth. To test these hypotheses we here used guppy (*Poecilia reticulata*) brain size selection lines with a pronounced negative association between brain and gut size and investigated feeding propensity, digestive efficiency (DE), and juvenile growth rate. We did not find any difference in feeding propensity or DE between large‐ and small‐brained individuals. Instead, we found that large‐brained females had slower growth during the first 10 weeks after birth. Our study provides experimental support that investment into larger brains at the expense of gut tissue carries costs that are not necessarily compensated by a more efficient digestive system.

Traditionally, research on within‐ and among‐species variation in brain size studies positive selection pressures, usually proxies of cognitive benefits, and negative selection pressures, for instance energetic costs (e.g., Jerison 1973; Aiello and Wheeler 1995; Dunbar 1998; Sol 2001; Isler and van Schaik 2006; Kotrschal et al. 2013a, b, 2014d). The assumption that larger brains are cognitively superior is supported by a number of studies (Sol et al. 2005; Kotrschal et al. 2013a; MacLean et al. 2014) and empirical research has also highlighted the “cost side” (Isler and van Schaik 2006) of larger brains (Aiello and Wheeler 1995; Isler and van Schaik 2006; Kotrschal et al. 2013a; Tsuboi et al. 2014). One of the key hypotheses in the research of brain costs, *the expensive tissue hypothesis*, states that due to the high energetic costs of developing and maintaining larger brains, they can only evolve under matching reductions in other costly organs (Aiello and Wheeler 1995; Aiello et al. 2001; Isler and van Schaik 2006; Kotrschal et al. 2013a; Tsuboi et al. 2014). The observation that prompted the conceptualization of the expensive tissue hypothesis was that, despite the three times larger brain in humans compared to the closely related chimpanzee, the basal metabolic rates per unit of body mass are very similar. This may be explained by the fact that humans have a substantially smaller gut, another very energetically costly organ in the vertebrate body (Aiello and Wheeler 1995; Aiello et al. 2001; Aiello and Wells 2002).

We recently used the guppy (*Poecilia reticulata*) in an artificial selection experiment to generate selection lines with large and small brains, which provide an experimental within‐species comparison of the costs and benefits of variation in brain size. In these lines, adult body size was not different, but relative (and absolute) brain size was 9% larger in the up‐selected lines already after two generations of selection. In support of the expensive tissue hypothesis, the difference in brain size was accompanied by substantial differences also in gut size. Gut size was substantially smaller (8% smaller in females and 20% smaller in males) in large brained lines than in small brained lines (Kotrschal et al. 2013a).

Given the high energetic expenditure of larger brains, how does the expensive tissue hypothesis explain how a smaller gut, the very organ that generates the available energy, can provide enough energy for an organism with a larger brain? According to Aiello et al. (2001), changes in diet quality, for instance to a diet based on more meat, may be one important way to accommodate increases in brain size with matching reductions in gut size. In support of this view, diet quality is also correlated with relative brain size in primates (Leonard and Robertson 1994). Here, we propose two additional nonmutually exclusive solutions to the “smaller gut but higher energy demands” problem of large‐brained individuals. Balancing of the higher energy demands of larger brains despite having a smaller gut could be achieved through (1) increasing food intake, or (2) increasing gut assimilation so that more energy is absorbed from a given amount of food. If food is abundant, increasing food intake should be relatively simple although it would require spending more time on feeding and less time on other important behavioral aspects, such as avoiding predation or attempting to mate (Brown 1999). Such behavioral changes could be under hormonal control, for instance through regulation of appetite, and either genetically hard‐wired or based on phenotypically plastic responses (e.g., Hill et al. 1991; Wynne et al. 2005; Suzuki et al. 2011; Zeng et al. 2012). Variation in gut assimilation levels, how effective the gut is at obtaining nutrients from the diet, is common at all taxonomic levels and can similarly be driven by both genetic factors and plasticity (Horn 1989; Horn et al. 2006; Karasov and del Rio 2007; Wagner et al. 2009; German et al. 2010; Kotrschal et al. 2014c; Sullam et al. 2015).

The higher energetic demands of larger brains might cause trade‐offs between brain size and early growth if increased food intake or better digestive efficiency (DE) do not compensate for a smaller gut. For instance, the most critical phase of human brain development is offset by reduced growth in early childhood as compared to other primates with smaller brains (Kuzawa et al. 2014). In fact, an early difference in growth rate is critical in organisms where survival and maturation is strongly determined by body size, such as in many aquatic species (Sogard 1997) and especially the guppy (Reznick 1982; Magurran 2005). Although adult body size is not different between the guppy brain size selection lines (Kotrschal et al. 2013a), juvenile growth rates may differ if individuals of different brain sizes have different energetic budgets that affect investment into biogenesis.

Here, we test these potential solutions to the “large brain–small gut” problem in the guppy brain size selection lines through experimental quantification of feeding propensity and gut assimilation efficiency. In relation to more indirect methods, such as for instance the quantification of gut flora (Sullam et al. 2015), these approaches more directly target the net effects of the smaller guts in large‐brained individuals. In addition, we measure the growth of large‐ and small‐brained individuals during the juvenile period. If the reduced gut size in large‐brained individuals is compensated for in appetite and/or gut efficiency, we predict that feeding propensity or gut assimilation efficiency will be greater in large brained individuals. If such compensations are adequate, we predict no differences in growth rates between selection lines. If compensations do not occur in feeding propensity or gut assimilation efficiency, or if such compensations are not sufficient, we predict that large‐brained individuals will have reduced early growth.

## Methods

### DIRECTIONAL SELECTION ON BRAIN WEIGHT

We examined the relationship between brain size and aspects of resource utilization, such as growth, feeding propensity, and DE, in laboratory lines of Trinidadian guppies that were artificially selected for large or small relative brain size (Kotrschal et al. 2012, 2013a). Briefly, these selection lines were generated using a standard bidirectional artificial selection design that consisted of two replicated treatments (three up‐selected lines and three down‐selected lines). Because brain size can only be quantified after dissection, we allowed pairs to breed at least two clutches first and then sacrificed the parents for brain quantification and used the offspring from parents with large or small relative brain size as parents for the next generation. More specifically, to select for relative brain size (controlled for body size), we selected on the residuals from the regression of brain size (weight) on body size (length) of both parents. We started with three times 75 pairs (75 pairs per replicate) to create the first three “up” and “down” selected lines (six lines in total). We summed up the male and female residuals for each pair and used offspring from the top and bottom 25% of these “parental residuals” to form the next generation parental groups. We then used the offspring of the 30 pairs with the largest residual sums for up‐selection and the 30 pairs with the smallest residual sums for down‐selection for each following generation. To avoid inbreeding, full‐siblings were never mated. See Kotrschal et al. (2013a) for full details on the selection experiment. The selection lines differ in relative brain size by 9% in F2 (Kotrschal et al. 2013a) and up to 14% in F3 (Kotrschal et al. 2014a), and adult body size does not differ between the lines (Kotrschal et al. 2014b). All fish were removed from their parental tanks after birth, separated by sex at the first onset of sexual maturation and then kept in single‐sex groups with a maximum density of five individuals in 3 L tanks containing 2 cm of gravel with continuously aerated water. We allowed for visual contact between the tanks. The laboratory was maintained at 26°C with a 12‐h light:12‐ dark schedule. Fish were fed a diet of flake food and freshly hatched brine shrimp six days per week. All measurements were done blindly because only running numbers identified tanks. We used several different groups of F3 animals for our assays. The groups were balanced over the sexes, the three replicates and the two brain size selection regimes. We used 72 new‐born individuals to investigate growth (of which 66 [(30 small/33 large brained] survived through the whole juvenile period). We then used the 49 of those that survived until two years of age (23 small‐brained/26 large‐brained individuals) for determination of adult body size. An additional 120 adult individuals were used for a test of feeding propensity. Finally, 180 individuals (60 females/120 males) were used for quantification of DE.

### FEEDING PROPENSITY

To determine the amount of food animals consume in one feeding bout when fed ad libitum, we kept 10 individuals together in bare 10 L tanks without gravel and an external filter system. During 10 days, we performed the following routine once per day: 1 h prior to feeding, we carefully cleaned the tank bottom and stopped the water flow created by the external filter. Then, we fed (previously counted) 800 (female tanks) and 100 (male tanks) sinking food pellets (0.5 mm diameter, New Life, Inc., FL), which could be consumed in one bite. Feeding usually stopped after 4 min. After 30 min, we removed and counted all uneaten pellets (pellets did not disintegrate). The 120 individuals in this experiment were kept in 12 tanks and assayed for 10 days. The number of observations was therefore 120.

### DIGESTIVE ASSIMILATION EFFICIENCY

Digestive assimilation efficiency is the ratio of energy assimilated to energy ingested (Flowerdew and Grove 1980), which indicates how much energy of the consumed food can be utilized. To determine DE of adults, we followed Kotrschal et al. (2014c). In brief, we manufactured food pellets from pulverized flake food and agar to which we added 3% chromic oxide (dry weigh) as inert marker. By determining the amount of chromic oxide in the feces, we could determine how much food the fish ingested (McGinnis and Kasting 1964). Note that we did not determine the fraction of energy in the feces from sloughed intestinal cells and mucus. Our measures therefore reflect “apparent DE,” which represents a minimum value for the energy taken from food (Throckmorton 1973; Kotrschal et al. 2014c). Guppies produce very little feces and males are much smaller than females; we therefore kept groups of 20 (males) or 10 (females) per subgroup (according to the two brain size selection regimes and the three replicates) each in similar bare 10 L tanks with external filter system as described above. Individuals were starved for one day to assure complete gut evacuation and in the evening fed pellets with chromic oxide. Prior to feeding, all feces and food remains were removed from the tanks. The next morning, we siphoned all feces from the tanks, dried them at 65°C for 24 h, and weighed them to the nearest 0.1 mg. We repeated this process for several days until all subgroups had produced at least 7 mg of feces (dry weight).

About 2–3 mg of the dried feces was used to determine Cr levels: microwave digestion of samples was performed in 7 mL 60:40 nitric acid and hydrogen peroxide mixture (HNO_3_, 65%; H_2_O_2_, 30%, both suprapure grade). Ninety‐three milliliters of ultrapure water was added, and the solution was filtered (0.45 μm), diluted 1:10 with 3% nitric acid, Co was added as internal standard, and Cr was analyzed by ICP‐CRI‐MS (inductively coupled plasma collision/reaction interface mass spectrometry) with a Varian 820‐MS (Varain, Santa Clara, CA) applying 80 mL/min hydrogen as collision gas to the sampler cone. The rest of the sample (12 mg [range: 2–28 mg]) was used to determine energy content; we determined energy content (J/g) from food and feces by bomb calorimetry using an adiabatic calorimeter (IKA 4000, Janke & Kunkel, Staufen, Germany) with a microbomb insert. Following Flowerdew and Grove (1980), DE was calculated as:

Efficency (%)=100×Cr2O3/ Energ y food Cr2O3/ Energ y faeces .

The amount of sample used in the analysis did not affect the calorimetry result (regression of initial sample weight vs. energy content: *R* ^2^ < 0.01). Although we collected feces of 180 fish over several days, the analysis requires relatively large sample amounts, which made it necessary to pool samples. The number of calorimetric measurements was therefore 12 (six from large‐brained and six from small‐brained animal groups).

### GROWTH

To determine juvenile growth parameters of large‐ and small‐brained animals, we reared animals in individual 3 L tanks from the day of birth until week 10 using the same feeding regime as during the artificial selection regime. This amount of food creates growth rates of approximately 60% of the maximal growth rates and the first 10 weeks represent the whole juvenile period of near‐linear growth (Reznick 1983). Weekly, we placed the animals in a 25 mm Petri dish with 5 mm of water and took dorsal pictures of the unsedated animals to determine standard length (SL, from the tip of the snout to the end of the caudal peduncle). We calculated the specific growth rate of SL (SGR; percentage of growth per day) per weekly period of each individual fish as

SGR = InS L1− InS L1 ag e2− ag e1×100,

where SL_1_, SL_2_, age_1_, and age_2_ are initial and final sizes and ages of two successive measurements (Ricker 1979). After two years, we also determined adult body size (SL) in the 49 individuals that survived to this age (23 small‐brained/26 large‐brained individuals) with a digital calliper.

### STATISTICAL ANALYSES

Male and female guppies show dramatic differences in adult body size and juvenile growth trajectories (Magurran 2005). We therefore analyzed juvenile growth rates separately for both sexes. We used general linear mixed effect models (GLMMs) with the weekly SGRs as dependant variable, measurement week as covariate, brain size selection regime as fixed effect, and replicate nested in brain size selection regime and individual ID as random effect. To additionally resolve at which specific time points large‐ and small‐brained individuals differed in body size, we ran individual GLMMs for every week with the respective SLs as dependant variable, brain size selection regime and sex as fixed effects, and replicate nested in brain size selection regime as random effect. For the analysis of adult body size we, used a GLMM analogous to the ones used for DE, but with adult SL as dependent variable. We analyzed food intake by performing a GLMM with the amount of pellets consumed per group and day as dependent variable; brain size selection regime and sex as fixed effects; and tank ID, day of experiment, and replicate nested in brain size as random effects. To investigate the effect of brain size on DE, we performed a GLMM with DE as dependent variable, brain size selection regime and sex as fixed effects, and replicate nested in brain size selection regime as random effect. All analyses were done with SPSS 22.0, SPSS, Inc., Chicago, IL.

## Results

### FOOD INTAKE

Animals ate on average 42.1 (±1.6, females) and 3.0 (±0.2, males) food pellets per feeding bout and individual, but brain size selection regime did not affect food intake (GLMM: brain size selection regime: *F*_1,7_ = 1.03, *P* = 0.34; sex: *F*_1,7_ = 69.80, *P* < 0.001; Table 1).

**Table 1:** Mean digestive assimilation efficiency (DE) and food intake in male and female guppies artificially selected for large and small brain size (±SE)

	Food intake	
	(food pellets)	DE (%)
Small brain females	37.4 ± 7.3	26.8 ± 2.0
Large brain females	46.7 ± 7.3	30.7 ± 4.3
Small brain males	2.9 ± 0.3	24.9 ± 7.3
Large brain males	3.1 ± 0.3	20.7 ± 4.7

### DIGESTIVE ASSIMILATION EFFICIENCY

Mean DE was 25.8% (±2.4) and artificial selection for relative brain size did not influence this efficiency; also males and females showed similar rates of DE (GLMM: brain size selection regime: *F*_1,4_ = 0.001, *P* = 0.98; sex: *F*_1,5_ = 3.34, *P* = 0.13; Table 1).

### GROWTH

#### Juvenile growth

**Figure 1:**
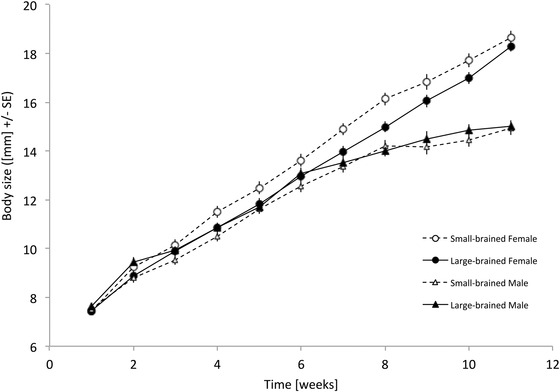
Growth curves of male and female guppies artificially selected for large and small relative brain size. Shown are the estimated marginal means of weekly GLMMs with replicate nested in brain size selection regime as random effect.

Large‐brained females grew slower than small‐brained females, especially early during the juvenile period (GLMM: brain size selection regime: *F*_1,55.2_ = 4.80, *P* = 0.033; measurement week: *F*_1,252.9_ = 158.36, *P* < 0.001, brain size selection regime × measurement week: *F*_1,252.9_ = 3.83, *P* = 0.051; Fig. 1). Males’ growth trajectories did not show a brain size dependent pattern (GLMM: brain size selection regime: *F*_1,258_ = 0.032, *P* = 0.86; measurement week: *F*_1,258_ = 232.27, *P* < 0.001; Fig. 1). Small‐brained females were significantly larger than large‐brained females in week 6 and tended to be larger in weeks 4, 5, and 7 (Table S1, Fig. 1).

#### Adult body size

Large‐ and small‐brained guppies kept in individual tanks for two years did not differ in body size (GLMM: brain size selection regime: *F*_1,46_ = 1.77, *P* = 0.19; sex: *F*_1,46_ = 230.43, *P* < 0.001).

## Discussion

Opposite to our predictions, we did not find any differences in feeding propensity or gut assimilation efficiency between the large‐ and small‐brained animals. Hence, we find no support for that the reductions in gut size associated with larger brains are compensated either through increased appetite or increased gut assimilation efficiency. Instead, we find our alternative prediction supported because juvenile growth rate is lower in the large‐brained lines, at least for females; we thereby identify yet another cost of larger brains that may affect fitness.

The lack of difference in feeding propensity and gut assimilation efficiency in our experiment suggests, in line with the expensive tissue hypothesis, that individuals with large brains and small guts do not have more efficient digestive systems, but that large brains are more likely to evolve in environments with higher quality food (Aiello et al. 2001). This assumption is corroborated by the fact that in the wild, predation pressure varies greatly between different populations of guppies (Endler 1980; Reznick 1982; Magurran 2005; Kotrschal et al. 2013a) and this variation in predation can be associated with variation in diet and gut function. For instance, individuals from high‐predation populations have been reported to be more selective for high‐quality dietary items (Bassar et al. 2010; Zandona et al. 2011), and to have shorter guts (Sullam et al. 2015) in relation to individuals in low predation populations. These observations suggest that the large‐brained guppy selection lines share their gut phenotypic and functional profile with wild high‐predation populations. The fact that population differences in gut morphology also exist in other fish species and in other taxa (e.g., Tracy and Diamond 2005; Horn et al. 2006; German et al. 2010) allows for future investigations of whether such associations between brain size, gut morphology, and dietary preferences are common in wild vertebrates. Moreover, the observation of similar basal metabolic rates between humans and chimpanzees despite their marked brain size difference underlies the original expensive tissue hypothesis (Aiello and Wheeler 1995). It is therefore necessary to investigate metabolic rates in the here used large‐ and small‐brained guppies to further clarify whether the relationships between brain and gut size is governed by similar mechanisms across vertebrates.

Although we do not detect any differences in our assay of feeding propensity and gut assimilation efficiency, behavioral differences between large‐ and small‐brained individuals may still allow for compensation for the smaller guts in large‐brained individuals. Behavioral strategies could ameliorate the decelerated juvenile growth rates we observed in large‐brained females. One possibility is that large‐brained individuals consume higher quality food. This again fits with the behavioral profile of animals from high‐predation populations because animals from such populations tend to consume more high‐quality food items (invertebrates), whereas animals from low predation tend to consume more low‐quality food items (detritus and algae; Bassar et al. 2010; Zandona et al. 2011). Another, not necessarily mutually exclusive possibility is that large‐brained individuals have a behavioral repertoire that allows them to feed at a high enough rate also in populations with high levels of predation. It was recently demonstrated that females with larger brains have 13.5% higher survival in a seminatural environment with predators, than small‐brained females (Kotrschal et al. 2015a). Moreover, large‐brained females spend less time on performing dangerous predator inspections in comparison to small‐brained females (van der Bijl et al. 2015). We therefore speculate that if large‐brained females are more efficient in avoiding predators, perhaps through higher cognitive abilities in perception and decision making, it may also provide them with more feeding opportunities in relation to small‐brained individuals in situations with high levels of predation. It stands clear that further experiments, particularly on diet selectivity and feeding behavior during predation threat, are needed before we can fully understand the association between brain size, gut size, and feeding behavior.

In our experimental set‐up, food availability was fixed. Animals could therefore not resort to the above‐described behavioral adaptations. This may explain why females of the large‐brained lines displayed slower juvenile growth than females from the small‐brained lines. But this finding also points toward an important net cost of having a larger brain and lends further support for the expensive tissue hypothesis. It is apparent that even under benign feeding conditions, such as those used in our experimental setup, the combination of a large brain and a small gut carries costs that limit juvenile growth. Even though the difference in relative brain size is evident already at birth in the guppy brain size selection lines (Kotrschal et al. 2013a), this pattern is comparable to how early childhood growth is hampered in humans during the early childhood phase when the energetic demands of brain development are greatest (Kuzawa et al. 2014). It is worth noting that the differences in growth between large‐ and small‐brained females were greatest during week 4–7 (Table S1). Whether this period is critical for brain development in the guppy is currently unknown, but will be investigated in future experiments. Another potential explanation for slower body growth in large‐brained females may lie within the potential time constraint of developing a larger brain, because a larger brain may simply require more time to develop. This mechanism has repeatedly been hypothesized to underlie the very long human adolescent period (Hawkes et al. 1998). Future studies will investigate this aspect by rearing animals on diets of different energetic content.

Reduced juvenile growth rate may negatively impact large‐brained animals in at least two ways. First, in fish and many other aquatic organisms, mortality usually decreases strongly with increasing body size, because the most important aquatic predators are gape‐size limited (Sogard 1997). The large‐brained animals may therefore suffer from increased juvenile predation. Second, slower growth may lead to a delay in sexual maturation, a life‐history trait of great importance for reproductive fitness across taxa (Stearns 1988; Roff 1992, 2002). These two points should thus potentially be added to the list of costs associated with evolving a larger brain (Isler and van Schaik 2006, 2009; Kotrschal et al. 2013a). The fact that in guppy females larger brains also confer survival benefits (Kotrschal et al. 2015a) may ameliorate the potential size‐dependent predation disadvantage of larger brained animals. Whether and to what extent this is the case will be clarified in future experiments. However, for the cost of delayed maturation, we will attempt a quantification based on the assumption that body size determines maturation in guppies (Magurran 2005). The youngest age at which females in the artificial selection experiment (Kotrschal et al. 2013a) give birth is 11.7 weeks (A. Kotrschal, pers. obs.). Given a three‐week gestation period (Houde 1997), females can thus be fertilized at 8.7 weeks. Visual inspection of the growth curves in Figure 1 suggests that the size difference of large‐ and small‐brained females at that time enables small‐brained females to start reproduction exactly one week (13.0%) earlier than large‐brained individuals.

We did not detect any difference in male juvenile growth between the large‐ and small‐brained lines. Guppies have a pronounced sexual size dimorphism with males being much smaller than females (Houde 1997), and contrary to males, females continuously grow throughout their life (Reznick 1983). Males therefore spend fewer resources on body growth than females. This sexual dimorphism can be explained by the fact that male reproductive success is generally more dependent on access to partners, whereas female reproductive success is more dependent on body size, because in viviparous fish larger females give birth to more offspring (Houde 1997). We suggest that it is this differential male and female investment into growth that has yielded the sex‐specific effect of encephalization on juvenile growth observed here. Potentially, growth is sacrificed for brain size in the large‐bodied females, whereas in the much smaller males the amount of resources invested into growth is not large enough to affect brain growth. This difference in energy requirements between males and females may thus explain why in males, but not in females, a relatively smaller gut can still supply sufficient energy for a relatively larger brain, even without increasing feeding propensity or DE.

The small quantities of feces produced by guppies and the relatively large requirements of sample volume for these analyses mean that the estimates of DE are based on a large number of individuals, but relatively few measurements. This may decrease statistical power and it might therefore be premature to conclude that large‐ and small‐brained animals do not show subtle differences in DE. However, the demonstration of decelerated growth in the large‐brained females renders a prominent role of altered DE in the evolution of brain and gut size unlikely.

In light of the many costs associated with increased encephalization, it stands clear that substantial benefits are required for larger brains to evolve. It has been suggested that such benefits mainly stem from increased ecologically relevant cognitive abilities associated with larger brains (e.g., Sol et al. 2005; Maklakov et al. 2011; Kotrschal et al. 2013a, 2014a, 2015a; Snell‐Rood and Wick 2013), or from increased attractiveness in individuals with larger brains (Miller 2000; Kotrschal et al. 2015b). Future studies need to focus on disentangling the positive and the negative effects on various aspects of organism biology before we can fully understand under what circumstances costly large brains may evolve in wild populations.

To conclude, we do not find any differences in feeding propensity or DE between guppies selected for large or small brains. Instead, we find that large‐brained females have slower growth than small‐brained females during the first 10‐week period after birth. Together, our results suggest that there is no compensation in either feeding propensity or DE in the large‐brained lines, at least not within the current experimental set‐up. Interestingly, our study identifies reduced growth rate as an additional cost of evolving and maintaining a larger brain and thus we conclude that large brains can only evolve in situations where the benefits of larger brains are substantial. High predation may be a factor of particular relevance (Kotrschal et al. 2015a; van der Bijl et al. 2015), and further empirical studies will be necessary to disentangle how preferences for different quality food items may differ in relation to brain size and predation pressure.

## DATA ARCHIVING

The doi for our data is doi:10.5061/dryad.ns141.

## Supporting information

Supporting **Table**S1
