# Supplementary material for: The effect of brain size evolution on feeding propensity, digestive efficiency, and juvenile growth
Source: Evolution. 2015 Oct 19;69(11):3013–20. doi: 10.1111/evo.12784 (PMC5057322; doi:10.1111/evo.12784)
Supplement: Supplementary file 1 — Supporting TableS1 [file EVO-69-3013-s001.docx]

**Supplementary Table 1**: Results of GLMMs comparing body size (SL) of large- and small-brained females reared individually. Replicate line nested in brain size selection regime is included as a random effect.

|  | df | F | P |
| --- | --- | --- | --- |
| Week 0 | 1/29.0 | 0.501 | 0.485 |
| Week 1 | 1/4.0 | 0.750 | 0.435 |
| Week 2 | 1/23.0 | 0.081 | 0.778 |
| Week 3 | 1/28.0 | 2.874 | 0.101 |
| Week 4 | 1/26.0 | 3.680 | 0.066 |
| Week 5 | 1/28.0 | 3.376 | 0.077 |
| Week 6 | 1/27.0 | 6.025 | 0.021 |
| Week 7 | 1/3.7 | 7.476 | 0.056 |
| Week 8 | 1/4.3 | 2.594 | 0.178 |
| Week 9 | 1/3.6 | 2.500 | 0.196 |
| Week 10 | 1/14.0 | 0.458 | 0.509 |
